# Relationship between family environmental factors and infant sleep: Family factors and infant sleep

**DOI:** 10.1002/imhj.70067

**Published:** 2026-01-07

**Authors:** Niina Palm, Pirjo Pölkki, Juha Hämäläinen, Anneli Kylliäinen, Outi Saarenpää‐Heikkilä, Pertti Töttö, Tiina Paunio, E. Juulia Paavonen

**Affiliations:** ^1^ Department of Social Sciences University of Eastern Finland Kuopio Finland; ^2^ The Finnish Institute for Health and Welfare (THL) Helsinki Finland; ^3^ Faculty of Social Sciences/Psychology Tampere University Tampere Finland; ^4^ Department of Paediatrics Tampere University Hospital, Wellbeing Services County of Pirkanmaa Tampere Finland; ^5^ Tampere Center for Child, Adolescent and Maternal Health Research, Faculty of Medicine and Health Technology Tampere University Tampere Finland; ^6^ Department of Psychiatry University of Helsinki Helsinki Finland; ^7^ Pediatric Research Center, Child Psychiatry University of Helsinki and Helsinki University Hospital Finland and Finnish Institute for Health and Welfare Helsinki Finland

**Keywords:** family environment, infant sleep, infant sleeping problems, parenting stress, parenting style, soothing methods

## Abstract

This study examines the relationship between infant sleep and parenting style, family climate, parenting stress, and soothing methods among Finnish families. Mothers completed questionnaires before birth and when their infants were 3 and 8 months old. Initially, 1667 mothers participated, with follow‐up responses from 1421 mothers and 1427 infants at 3 months and 1298 mothers and 1302 infants at 8 months. A cross‐sectional data analysis was conducted using linear regression to examine the four sleep‐related outcome variables, and parallel multivariable regression models were built using backward stepwise selection. Predictors were selected based on statistical significance in the pre‐screening regression analyses. Models controlled for maternal age at birth, infant gender, older siblings, breastfeeding, and maternal education. The findings indicate that higher parenting stress, and active and passive physical soothing styles are associated with more problematic sleep. A higher control showed a weak association with higher sleep problem severity scores, whereas a more active recreationally oriented family climate was weakly associated with shorter sleep onset latency. This study contributes to the current body of research on children's sleeping problems and the family environment, and it would be beneficial for social and healthcare services to take these findings into account.

## 1 INTRODUCTION

Sleeping problems are common among families with small children. It has been estimated that up to 25%–50% of 6–12‐month‐old babies wake up frequently at night or have problems falling asleep (Mindell & Owens, 2010; Paavonen et al., 2020). Sleeping disturbances are one of the infant regulatory problems, and they often coexist with prolonged crying—another regulatory problem (Singh et al., 2021). These regulatory problems are often corrected over time, but not always (Scher et al., 2005).

Different kinds of risk and protective factors in a child's psychosocial environment are known to influence the child's sleep. Usually, these psychosocial factors influence a child's sleep through the interplay between the caregiver and the child (Mindell & Owens, 2010). Moreover, psychosocial and cultural factors play a significant role in sleep development (El‐Sheikh & Kelly, 2017), and contribute to individual differences in sleep alongside genetic (Viktorsson et al., 2025) and biological factors (Webb et al., 2024). General habits and the prevailing culture influence parental opinions, for example, about where and how children should sleep and what is considered normal sleep (Pajulo et al., 2012; Jenni & O'Connor, 2005; Blunden et al., 2011). What is labeled as a sleep problem may sometimes reflect cultural variations in sleep practices rather than an actual sleep problem.

In the present study, we wanted to find out more about the associations between these psychosocial, family‐related factors, and infant sleep. The family‐related factors chosen for this study concern both the context of the parent–child relationship, including parenting style, family climate, parenting stress, and parental sleep‐related behaviors—parents’ soothing methods. Throughout this paper, we use the term sleep/sleeping problems to refer broadly to parent‐reported issues, such as frequent night wakings, difficulties falling asleep, and perceived sleep disturbances, or sleep problems measured with objective measurements. Although the literature uses various terms (i.e., sleep difficulties, sleep disturbances), we use sleep problems consistently for clarity.

Parenting styles encompass a set of parental attitudes, beliefs, and behaviors conveyed to a child through their interaction with the parent (Kivijärvi et al., 2009; Philips et al., 2014), and have a significant impact on a child's early socioemotional development (Vasiou et al., 2023; Vilhula, 2007) and mental health (Fadlillah et al., 2020; Wang et al., 2024). However, to our knowledge, there are no studies on parenting styles and infant sleep development. Existing studies (Meijer et al., 2001; Spilsbury et al., 2005; Johnson & McMahon, 2008; Tyler et al., 2019; Hall et al., 2007; Jin & Chen, 2024) on the influence of parenting style on child sleep have focused on older children and reported somewhat varying results. This may be due to the diversity of definitions of parenting style (Philips et al., 2014). Our study draws on the classical model of parenting presented by Baumrind (1966), which recognizes three types of parenting style: authoritative, authoritarian, and permissive. An authoritative parent encourages the child to engage in dialogue, explains the reasoning behind their actions, respects the child's individual goals, and simultaneously maintains expectations of compliance (Kivijärvi et al., 2009; Baumrind, 1966). An authoritarian parent controls the child and is even willing to use physical punishment, refusing to negotiate or respect the child's goals if they contradict the parent's. A permissive parenting style means that the parent avoids being controlling and does not discipline the child, even when the child exhibits challenging behavior, and may feel uncertain about how to interpret and respond to such conduct.

**KEY FINDINGS**

- Mothers’ higher parenting stress and active and passive physical soothing styles were associated with more problematic infant sleep, and there were indications that a control‐oriented family climate might be linked to increased sleep problems in infants.
- An active, recreationally oriented family climate showed a weak connection with shorter sleep onset latency.
- Permissive, authoritative, and authoritative parenting styles, and other types of family climates were not found to be associated with infant sleep quality.

**STATEMENT OF RELEVANCE**

Sleep is a critical factor in infant and parental health and well‐being, as well as infant development. This study adds to the limited knowledge base on the importance of the family environment for infant sleep. For social and health services to provide effective support for parents seeking help with infant sleeping problems, it is essential to know which factors are most connected to these problems.

Previous—mainly cross‐sectional—studies have reported an association between a relaxed, permissive parenting style and children's sleep problems (Tyler et al., 2019; Meijer et al., 2001). Hall et al. (2007) found in their longitudinal study that lax parenting of 2‐year‐old children predicted sleeping problems when children were 3 years old. Also, Shetty et al. (2022) reported that higher parenting laxness was associated with more severe sleep problems with 2–10‐year‐old children when the bedtime reactivity of parents was higher. Tyler et al. (2019) discovered that a permissive parenting style mediated the relationship between parental distress and pre‐school‐aged children's sleeping problems. Rodriguez et al. (2025) also studied preschool‐aged children and found that the mother's history of anxiety disorders combined with a more permissive parenting style when the child was 3 years old predicted more sleeping problems at age 6.

Some studies have recognized active and authoritative parenting style as a protective factor for children's sleep (Spilsbury et al., 2005; Johnson & McMahon, 2008; Jin & Chen, 2024). Jin & Chen (2024) discovered that a low‐authoritative parenting style was associated with severe sleep problems in 2–5‐year‐old Chinese children, but only in families with one child. The authors suggest that the reason for this association may lie in the secure parent–child relationship enabled by authoritative parenting, which promotes psychological well‐being and thereby supports better sleep and emotional regulation (Jin & Chen, 2024). Having a sibling seems to promote sleep in a way comparable to sleep aids (Jin & Chen, 2024).

Previous findings regarding especially authoritarian or similar parenting styles and children's sleep have been inconsistent. Most of these studies have found connections between such a parenting style and poorer infant sleep. Philips et al. (2014) found that 6–12‐year‐old children sleep less when the parent uses a more coercive and controlling parenting style. “Harsh parenting,” which means acts of hostility and physical control, has also been associated with insufficient sleep duration and night wakings in 2–6‐year‐old children from lower socioeconomic backgrounds (Kelly et al., 2021). In contrast, Rodriguez et al. (2025) found that mothers’ more authoritarian parenting style when the child was 3 years old was associated with fewer sleep problems at age 6. The reason for this might be that preschool‐aged children require strict boundaries and more external control during bedtime (Rodriguez et al., 2025). However, Tyler et al.’s (2019) study showed no evidence of a relationship between authoritarian or authoritative parenting styles and preschool‐aged children's sleep problems.

Also, a connection between an overprotective parenting style and sleep problems in young children has been identified, although this style is not included in the model used in our study. According to Zaidman‐Zait & Hall (2015), extended night waking at 29 months was associated with parental overprotectiveness—defined as parenting style or practice with excessive concern for the child's safety and protection. Overprotectiveness scores were measured when the child was 5 months old and were highest among parents whose children exhibited excessive night waking at 29 months. Pizzo et al. (2022) reached a similar conclusion in their study reporting maternal overprotectiveness to be associated with impaired sleep in children aged 2 to 6 years. Therefore, we hypothesize that an authoritative parenting style is related to better and permissive and authoritarian parenting styles to poorer sleep quality in infants.

Studies have demonstrated that family climate plays an important role in children's development and well‐being (Moos & Moos, 2009, review). Family climate is a somewhat abstract concept that can be conceptualized in various ways, and our study utilizes the family climate framework developed by Moos & Moos (2009), who conceptualized the family climate in three main dimensions, each comprising specific subscales on the Family Environment Scale (FES; Moos & Moos, 2009): Family system maintenance—organization and control; social relationships—cohesion, expressiveness, and conflict; and personal growth—independence, achievement orientation, intellectual‐cultural orientation, active‐recreational orientation, and moral‐religious emphasis.

Only a few studies have employed the Family Environment Scale in the context of child sleep research, and to our knowledge, none have done so in the context of infant sleep. In Bates et al.’s (2002) study, the FES subscale of “cohesion” was part of the family management construct when studying how differences in daily patterns of sleep were related to the adjustment of 4–5‐year‐old children. Cohesion refers to the degree of commitment, help, and support that family members provide to one another (Moos & Moos, 2009). However, cohesion was not directly associated with children's sleep patterns (Bates et al., 2002). FES's cohesion dimension has also been utilized in Hairston et al.’s study (2016) alongside conflict dimension. The purpose of the study was to examine whether children's sleep characteristics mediate the relationship between resiliency and behavioral problems in children aged 7–13 with varying levels of familial risk for alcoholism. Sense of cohesion and conflict were assessed through child reports, and the results showed that greater cohesion was associated with a longer total sleep time, while higher conflict was linked to shorter sleep duration (Hairston et al., 2016). Family conflict measured with FES has been linked to sleep disturbances among adolescents (Zhang et al., 2018). Given the scarce and fragmentary prior evidence, we hypothesize that among family climate factors, a higher conflict in the family is associated with more problematic infant sleep.

The third factor describing the family environment is parenting stress. Several cross‐sectional studies have found an association between infant sleep and maternal parenting stress (Meltzer & Mindell, 2007; Doo & Wing, 2006; Hodge et al., 2013; Zengin Akkus & Bahtiyar‐Saygan, 2022; Tyler et al., 2019). There is also evidence of a link between paternal stress and children's sleep problems (Thome & Skuladottir, 2005; Bernier et al., 2013; Millikovsky‐Ayalon et al., 2015). Parents’ stressful situations in life due, for example, to unemployment or illness, can be stressful for children, possibly manifesting as sleeping problems (Sadeh, 2001). Stress can influence parents’ interactions with their children, often making them less sensitive toward them. Infants have been shown to be very sensitive to their parents’ emotional circumstances and to absorb and react to their caregivers’ situations (Sadeh, 2001). Parents with lower levels of stress are likely to be more emotionally available, providing children with a sense of emotional security and a stable family environment that promotes better sleep quality (Bernier et al., 2013). However, there is limited empirical evidence on this kind of directionality between child sleep and parenting stress (Tikotzky et al., 2021). Surprisingly, Tikotzky et al.’s (2021) longitudinal findings, based on latent trajectory analyses of 226 Israeli mothers and infants, do not support the assumption that maternal emotional distress symptoms (including parenting stress, measured with the Parenting Stress Index by Abidin, 1995) cause infant sleep problems over time. Instead, they found that distressed and (especially) mothers with parenting stress symptoms are more likely to experience their 18–month‐old infant's sleep as problematic while actigraphy data did not reveal this association. Based on the majority of previous research findings, we hypothesize that parenting stress is related to more sleeping problems in infants.

Soothing methods, which parents use while putting their infants to sleep, constitute the fourth beneficial family environmental factor. The link between soothing methods and infant sleep is well documented. Excessive use of active physical comforting (i.e., holding, rocking, feeding) and reduced use of strategies to encourage autonomy have been associated with infant sleeping problems in both cross‐sectional (Morrell & Cortina‐Borja, 2002; Sadeh et al., 2010, 2009; Adams et al., 2022) and a few longitudinal studies (Morrell & Steele, 2003; Henderson et al., 2020; Matzliach et al., 2025). The understanding is that infants fail to develop their own soothing skills if their parents use overly active soothing methods and neither limit their involvement nor encourage self‐soothing (Sadeh et al., 2010; Zreik et al., 2021; Adams et al., 2022). Infants who fall asleep independently tend to wake less frequently and are more likely to self‐soothe during nighttime awakenings (Adams et al., 2022; Matzliach et al., 2025; Camerota et al., 2019). Adams et al. (2022) used actigraphy to measure 6‐ to 24‐week‐old infants’ sleep, and found that the use of low‐stimulus soothing strategies was associated with more frequent self‐soothing behaviors. In other words, when parents used more low‐stimulus soothing strategies, there was a greater proportion of infant‐only wake bouts at night. We hypothesize that an active physical soothing style is connected to poorer sleep quality.

Understanding the circumstances that best support infants’ sleep development is highly significant. Parents often consider their infants’ sleep problematic and seek support to improve their sleep quality. The prevalence of parent‐reported sleeping problems has been found to be as high as 21.9% at the age of 3 months and 39.4% at the age of 8 months (Paavonen et al., 2020). Sleep is crucial for both infant and parental health and well‐being. Especially when infant sleeping problems are persistent, they may cause parental exhaustion and other health problems, impair the infant's mental health, and have a negative effect on infant socioemotional development (Singh et al., 2021; Mindell et al., 2017). Sleeping problems can also increase the risk of mental health problems and behavioral problems later in childhood (Cook et al., 2020; Scher et al., 2005).

Supporting healthy sleep in children is crucial, as it can promote mental health both in infancy and later in life (Kocevska et al., 2023). Therefore, identifying family environmental factors that influence a child's sleep is particularly important, as environmental aspects are more amenable to change than targeting the underlying biology, which also affects sleep (Kocevska et al., 2023). Moreover, for social and health services to provide impactful and comprehensive support to these parents, practitioners must be aware of the factors that are linked to and may influence sleep problems in infants. Thus, the overall goal of our study is to obtain a comprehensive picture of the relationship between the family environment, as reported mainly by mothers, and infant sleep at the age of 8 months from a large population‐based sample. Specifically, its aim is to explore which factors of the family environment (including parenting styles, family climate, soothing styles, and parenting stress), and which of the dimensions of chosen factors, increase infants’ sleeping problems. The factors were chosen based on previous studies, and our aim was to test and complement previous findings. Our study was carried out in Finland, which is a Nordic welfare state with a good family service system and strong support for families with children.

## 2 PARTICIPANTS AND METHODS

We used data from the CHILD‐SLEEP study, a longitudinal birth cohort. It focused on examining the early development of sleep and the circadian rhythm, as well as the value of sleep for infants’ health and well‐being (Paavonen et al., 2017). The study was conducted in cooperation with the Finnish Institute of Health and Welfare, the Pirkanmaa Hospital District, the University of Eastern Finland, the University of Helsinki, and Tampere University. The birth cohort was drawn from infants born between April 2011 and March 2013 in the Pirkanmaa Hospital District.

**FIGURE 1:**
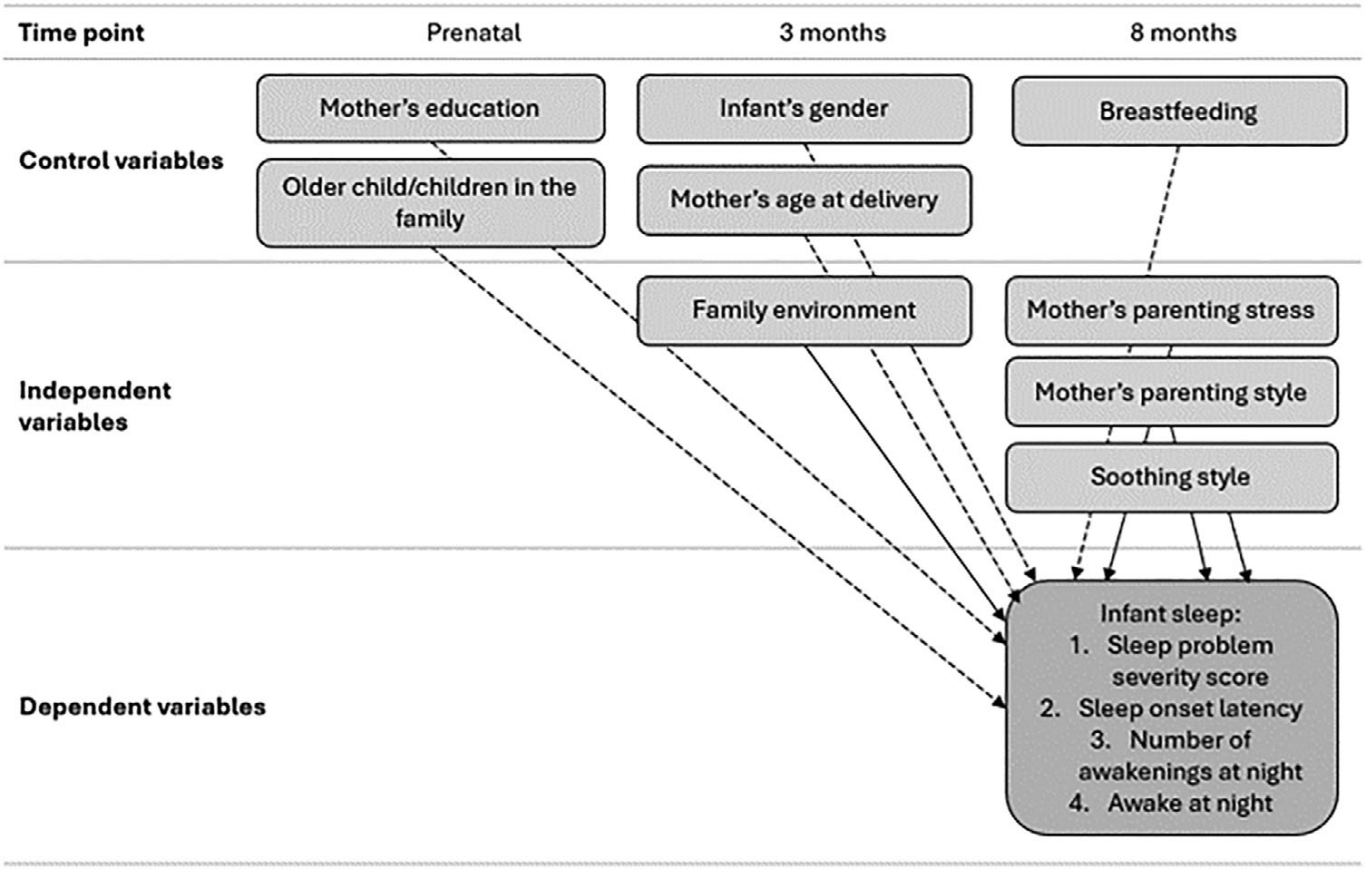
An overview of the study design.

The data used in this paper were collected in three phases. Families were invited to join the study during routine visits to maternity clinics. There, prenatal questionnaires were handed out during the 32nd week of pregnancy. The follow‐ups were conducted when the infants were 3 and 8 months old. At both time points, the participants were asked to complete questionnaires for both mothers and infants. In this study, we used the mothers’ data at the prenatal, 3‐month, and 8‐month time points and the infants’ data at the 8‐month time point. The study protocol was approved by the Ethics Committee of Pirkanmaa Hospital District (R11032), and all subjects provided written informed consent. An overview of the study design, variables, and time points is provided in Figure 1.

The sample included 1667 mothers at the prenatal time point, 1421 mothers and 1427 infants at the 3‐month time point, and 1298 mothers and 1302 infants at the 8‐month time point. Twenty cases were excluded due to severe infant illness. In the case of twins, only one twin was included in the analysis (5 excluded cases). At the 8‐month time point, 71.2% of the questionnaires for infants were filled out by mothers or an adult in the maternal role, 1% by fathers or an adult in the paternal role, and 27.8% by both parents. There were no significant differences in the prevalence rates of reported sleep problems based on who completed the questionnaire.

Attrition analyses (*t*‐tests and chi‐square tests) were conducted to examine differences between participants who discontinued after the prenatal or 3‐month time point and those who remained in the study. Dropout was associated with significantly higher prenatal levels of depressive symptoms (CES‐D; *M *= 5.71) compared to completers (*M *= 4.96); *t*(522.6) = −3.3, *p *= .001), to lower educational attainment, *χ^2^* (1) = 13.95, *p *< .001, and having more children (*M *= .95) compared to those who completed the study (*M *= .71); *t*(1524) = −4.48, *p *< .001.

*Sleep measures*. The infant sleep questionnaire (ISQ; Morrell, 1999; *α* = .50) was used to define infant sleeping problems at the 8‐month time point. We used the sleep problem severity score, a composite score ranging from 0 to 38 (where 0 indicates no sleeping problems at all and 38 represents severe sleeping problems) computed from six questions concerning settling to sleep, waking up at night, resettling, and sleeping in the parents’ bed (i.e., “How long does it usually take to settle your baby to sleep?”, “Do you think your baby has sleeping problems?”). The ISQ has well‐documented concurrent validity (Morrell, 1999), good reliability data, sensitivity, and specificity for identifying sleep problems, and has been assessed as a well‐established sleep measure (Lewandowski et al., 2011).

We also used single variables from the brief infant sleep questionnaire (BISQ; Sadeh, 2004) as the dependent variables at the 8‐month time point. The variables used were 1) sleep onset latency (“How long does it take to put your baby to sleep?”), 2) number of awakenings at night (“How many times does your child wake up at night?”), and 3) duration of being awake at night (“How long does your child stay awake between 10 pm and 6 am?”). All of these were open‐ended numerical questions, and the responses to the first and latter questions were given in hours and minutes. Logarithmic transformation was used to normalize the distribution of BISQ measures. We use the term sleeping problems as a general descriptor for all previously mentioned sleep‐related difficulties reported by parents. All sleep‐related outcome variables were treated as continuous measures. We did not apply clinical cutoffs or categorical definitions of sleep problems.

*Parenting style*. The parenting styles and dimension questionnaire (PSDQ; Robinson et al., 2001) includes 62 statements (i.e., “I joke and play with our child”, “I spoil our child”) rated on a five‐point Likert scale from “never” to “always,”. Its purpose is to measure parenting styles that originated from Baumrind's (1966) global typology and the dimensions and internal structures within the typologies. The PSDQ was not used as a whole because it also includes items that concern the parenting of older children. To avoid participant confusion, 24 items that were not suitable for parents of infants (i.e., “I encourage our child to talk about the child's problems”) were not included in the questionnaires. This resulted in a 38‐item scale at the 8‐month time point. Three composite scores were formed from those variables, one for each parenting style dimension. Higher scores indicated a stronger representation of the corresponding parenting style. Cronbach's alpha values were .78 for the authoritative subscale, .73 for the authoritarian subscale, and .53 for the permissive subscale.

*Family climate*. The FES (Moos & Moos, 2009) measures a person's perceptions of their family environment and the family's social climate. It consists of 90 items, each answered with a “Yes” or “No” option (i.e., “Family members rarely lose their temper”, “Punctuality is considered very important in our family”). These items were originally reported to produce a 10‐dimensional factor structure. Several studies have used the FES to describe the impact of the family climate on child development (Moos & Moos, 2009), and some have also tried to replicate the factor structure of the FES without success, which was also the case in our sample. To get a unidimensional subscale for each conceptual dimension for FES, we had to drop three items with low communalities from each subscale. The unfit items were identified with confirmatory factor analysis using a mean and variance adjusted weighted least squares estimator in MPLUS. After testing the measurement models, we computed a composite score and calculated McDonald's omega coefficient as a reliability estimate for each dimension. Higher scores indicated a stronger representation of the corresponding dimension. The reliabilities of the subscales were: cohesion (.936), expressiveness (.824), conflict (.848), independence (.533), achievement orientation (.742), intellectual–cultural orientation (.725), active‐recreational orientation (.692), moral–religious emphasis (.909), organization (.761), and control (.686). The FES was applied at the 3‐month time point.

*Parenting stress*. Parenting stress was measured using the parenting stress inventory (PSI; Terry, 2001) at the 8‐month time point. The PSI (*α* = .87) includes 23 statements about challenges in parenthood, for example regarding the sense of losing independence and reduced couple time. Participants were asked to evaluate the frequency and severity of the challenges they had experienced over the past two weeks. The five‐point scale ranged from “No, I have never experienced it” to “Yes, I have experienced it very seriously.” A composite score was formed from these 23 items, and higher scores indicated a stronger representation of parenting stress.

*Soothing methods*. We used the parental interactive bedtime behavior scale (PIBBS; Morrell & Cortina‐Borja, 2002), which consists of 21 items describing different kinds of parental behaviors used in settling infants to sleep. The parents were asked to rate on a five‐point scale (from “never” to “very often”) whether they used the soothing methods described at the 8‐month time point. This generated scores from 1 to 5 for each item, where a lower score means rarer use of the described strategy. Five subscales were formed from the items describing different strategies. “Active physical comforting” (*α* = .58) means rocking in arms and cuddling, giving a feed, carrying the infant in arms around house, and other active and physical behaviors, while “encouraging infant autonomy” (*α* = .18) consists of leaving the infant to cry, playing music tape or a musical toy, and offering the infant a special toy or cloth. “Settling by movement” (*α* = .38) means settling with the help of walks in pram or buggy and car rides, while “passive physical comforting” (*α* = .59) consists of standing next to the crib without picking up the infant and laying with infant next to their crib. Finally, “social comforting” (*α* = .499) means talking softly to the child, singing a lullaby, reading a story, and playing with the infant. As the reliability estimates of some of the soothing style scales were low, we included only those with Cronbach's alpha over .5 in our regression analyses. According to the methodological literature, an alpha value above .5 may be considered acceptable in certain contexts, especially with short scales or exploratory research.

The descriptive statistics and correlations of the measures were calculated. To examine associations between family‐related predictors and sleep outcomes, we first ran a series of preliminary regression analyses with one predictor of interest along with relevant covariates (mother's education, older child/children in the family, infant's gender, mother's age, and breastfeeding). The predictors were three parenting‐style variables, ten family climate variables, parenting stress, and two soothing‐style variables. Afterward, multivariable analyses were performed to find out which predictors have the strongest effects in the presence of other predictors. We included in the multivariable models those predictors that were statistically significant (p < .05) in the preliminary (pre‐screening) regression analyses. In the multivariable analyses, we applied a backward stepwise selection method, starting with the full model and sequentially removing predictors until further elimination significantly reduced model fit. The analyses were conducted separately for each of the four sleep‐related outcome variables. The dependent variables were the sleep problem severity score and single BISQ variables: sleep onset latency, the time spent awake at night (i.e., how many minutes the infant is awake between 10 pm and 6 am), and the number of night awakenings (i.e., how many times the infant wakes up during the night). The threshold for statistical significance was defined as *p* < .05, as is the established practice.

## 3 RESULTS

The descriptive statistics of variables used in the analysis are presented in Table 1, and their intercorrelations in Table 2.

**TABLE 1:** Descriptive statistics.

	Time point	Mean (SD)	*N* (%)	Min‐max	Skewness	Kurtosis
**Infant's gender**	3 mo					
Female			609 (47.7)			
Male			668 (52.3)			
**Breastfeeding**	8 mo					
Yes			819 (65.1)			
No			439 (34.9)			
**Mother's age at delivery**	3 mo	30.66 (4.499)	1628	17–48	.208	.135
**Mother's education**	pre					
High (university, polytechnic, or institute)			1148 (69.7)			
Low			498 (30.3)			
**Older child/children in the family**	pre					
Yes			808 (52.9)			
No			718 (47.1)			
**Infant sleep**	8 mo					
Sleep problem severity score		13.336 (5.893)	978	1–32	.181	−.120
Sleep onset latency		22.542 (18.016)	1237	0–300	4.332	48.322
Number of awakenings at night		2.3872 (1.694)	1215	0–12.50	1.686	5.167
Awake at night		24.855 (31.444)	1149	0–450	4.950	45.182
**Mother's parenting style**	8 mo					
Authoritative		4.205 (.400)	1276	2.08–5	−.580	.648
Authoritarian		1.651 (.358)	1275	1–3.17	.336	.008
Permissive		1.917 (.296)	1275	1–3.08	.300	.457
**Mother's parenting stress**	8 mo	39.373 (7.940)	1181	24–80	.938	1.610
**Soothing style**	8 mo					
Active‐physical		16.413 (4.460)	1230	6–30	.059	−.341
Passive‐physical		4.7223 (2.188)	1253	2–10	.721	−.112
**Family environment**	3 mo					
Cohesion		5.538 (.957)	1372	0–6	−2.983	10.504
Expressiveness		5.356 (.972)	1362	0–6	−1.980	4.832
Conflict		1.733 (1.333)	1360	0–6	.689	−.086
Independence		4.530 (1.086)	1342	0–6	−.646	.294
Achievement		1.595 (1.132)	1344	0–6	.475	.063
Intellectual		3.449 (1.476)	1360	0–6	−.131	−.732
Active‐recreational		3.042 (1.535)	1348	0–6	−.132	−.846
Moral		2.068 (1.550)	1346	0–6	.799	.188
Organization		4.408 (1.397)	1354	0–6	−.762	−.076
Control		3.341 (1.320)	1364	0–6	−.057	−.689

**TABLE 2:** Intercorrelations of variables used in the analysis.

	1.	2.	3.	4.	5.	6.	7.	8.	9.	10.	11.	12.	13.	14.	15.	16.	17.	18.	19.	20.	21.	22.	23.	24.	25.
1. Severity score^1^	1																								
2. Sleep onset^2^	.423***	1																							
3. No. of awakenings^2^	.492***	.081**	1																						
4. Awake at night^2^	.297***	.179***	.225***	1																					
5. Cohesion^3^	−.097**	−.068*	−.044	−.024	1																				
6. Expressiveness^3^	−.091**	−.088**	−.065*	−.041	.461***	1																			
7. Conflict^3^	.093**	.038	.074*	−.004	−.348***	−.128***	1																		
8. Independence^3^	−.078*	−.007	−.064*	−.008	.250***	.212***	−.217***	1																	
9. Achievement^3^	−.008	.056	−.026	.029	−.077**	−.043	.041	.184***	1																
10. Intellectual^3^	.025	−.005	.045	−.007	.125***	.175***	.013	.092**	.050	1															
11. Active−recr.^3^	.009	−.067*	.042	.026	.147***	.174***	−.056*	.132***	.104***	.370***	1														
12. Moral^3^	.065*	.033	.053	−.09	.004	.022	.065*	−.051	.058*	.124***	.137***	1													
13. Organization^3^	−.095**	−.091**	−.027	−.037	.244***	.117***	−.173***	.262***	.031	.031	.099***	.040	1												
14. Control^3^	.086**	−.047	.049	−.057	−.166***	−.067*	.389***	−.194***	.035	.042	.015	.163***	.011	1											
15. Authoritative^4^	−.042	−.058*	−.023	.026	.189***	.212***	−.201***	.230***	.057	.134***	.085**	−.061*	.128***	−.080**	1										
16. Authoritarian^4^	.024	−.021	.047	−.065*	−.099***	−.069*	.278***	−.141***	−.008	.042	−.039	.031	−.089**	.312***	−.144***	1									
17. Permissive^4^	.111**	.056*	.081**	.035	−.147***	−.140***	.129***	−.187***	−.028	−.055	−.115***	−.061*	−.210***	−.024	−.374***	.058*	1								
18. Parenting stress^5^	.256***	.189***	.195***	.173***	−.294***	−.212***	.190***	−.130***	.130***	−.061*	−.096**	−.018	−.168***	.094**	−.188***	.132***	.248***	1							
19. Active−physical^5^	.359***	.252***	.192**	.108	−.062*	−.077**	.053	−.021	.107***	.079**	−.013	.063*	−.105***	.009	−.005	.028	.132***	.178***	1						
20. Passive−physical^5^	.165***	.119***	.097***	.028	−.052	−.073*	.040	.055	−.003	.026	.026	.023	−.022	.005	.014	.022	.028	.094**	.092*	1					
21. Mother's education	.146***	.012	.058**	.027	.127***	.067*	−.023	.028	−.065*	.204***	.094**	.007	.056*	−.031	.022	.089**	.001	−.012	.012	−.026	1				
22. Mother's age	.064*	.044	.007	.022	−.008	−.014	.048	−.008	−.131***	.172***	.062*	−.003	.070*	.100***	.014	.142***	−.041	−.066*	.073*	.036	.334***	1			
23. Older children	.079*	−.037	.067*	−.025	−.116***	−.058*	.345***	−.290***	−.058*	.057*	−.025	.104***	−.088**	.538***	−.155***	.424***	.058*	−.030	.036	−.042	−.014	.267***	1		
24. Infant's gender	.037	.038	−.006	.017	.049	−.004	−.026	.005	−.011	.051	.024	−.059*	−.002	−.039	.001	.006	.014	.007	−.001	.040	.063*	.035	−.023	1	
25. Breastfeeding	.267***	.051	.175***	.047	.012	−.020	.047	−.091**	−.050	.151***	.072*	.124***	−.044	.017	−.022	.094**	−.003	.031	.187**	.033	.226***	.114***	.102**	.009	1

Abbreviations: ^1^ISQ, infant sleep questionnaire; ^2^BISQ, brief infant sleep questionnaire; ^3^FES, Family Environment Scale; ^4^PSDQ, parenting styles and dimensions questionnaire; ^5^PSI, parenting stress inventory; ^6^PIBBS, Parental Interactive Bedtime Behaviour Scale.

***p < .001, **p < .01, *p < .05

The regression results for the sleep problem severity score are shown in Table 3. In preliminary regression models controlling for maternal age, education, number of children, breastfeeding, and previous child or children in the family, seven predictors showed statistically significant associations with more problematic sleep. Variables found to be significant, along with the control variables, were included in the multivariable analysis. An analysis with the backward stepwise method was performed, starting with the previously mentioned control variables and seven predictors: parenting stress, active and passive physical soothing styles, permissive parenting style, and some FES dimensions (cohesion, expressiveness, conflict, and organization). The final model retained six predictors. Parenting stress (*β* = .185, *p* < .001), active (*β* = .277, *p* < .001) and passive (β = .119, *p* < .001) physical soothing styles, control (*β* = .066, *p* = .023), breastfeeding (*β* = .179, *p* < .001), and mother's education (*β* = .109, *p* < .001) were connected to more problematic sleep. The final model was statistically significant (F(6, 904) = 46.839, *p* < .001), and the model's coefficient of determination was 23.2%.

**TABLE 3:** Results from pre‐screening regression analyses (one main predictor with covariates) and final multivariable regression model for sleep problem severity score (ISQ).

	Regression with one main predictor^1^					Multivariable regression^2^			
Predictor	B (SE)	*β*	*t*	*p*	*R* ^2^	B (SE)	*β*	*t*	*p*
Parenting stress	.187 (.023)	.252	8.186	**<.001**	.146	.137 (.022)	.185	6.215	<.001
Active−physical soothing style	.429 (.041)	.325	10.598	**<.001**	.185	.366 (.040)	.277	9.084	<.001
Passive−physical soothing style	.439 (.085)	.163	5.169	**<.001**	.110	.320 (.079)	.119	4.055	<.001
Authoritative parenting style	−.444 (.476)	−.030	−.933	.351	.084				
Authoritarian parenting style	−.633 (.581)	−.039	−1.089	.276	.084				
Permissive parenting style	2.153 (.632)	.108	3.406	**<.001**	.095				
Cohesion (FES)	−.677 (.198)	−.110	−3.421	**<.001**	.095				
Expressiveness (FES)	−.547 (.193)	−.090	−2.832	**.005**	.091				
Conflict (FES)	.322 (.150)	.073	2.153	**.032**	.088				
Independence (FES)	−.249 (.181)	−.046	−1.375	.169	.085				
Achievement (FES)	.064 (.167)	.012	.385	.700	.083				
Intellectual (FES)	−.145 (.131)	−.036	−1.099	.272	.084				
Active−Recreational (FES)	−.060 (.123)	−.016	−.483	.629	.083				
Moral (FES)	.117 (.123)	.031	.956	.339	.084				
Organization (FES)	−.361 (.135)	−.086	−2.669	**.008**	.090				
Control (FES)	.349 (.169)	.078	2.066	**.039**	.088	.298 (.130)	.066	2.272	.023
Breastfeeding						2.213 (.376)	.179	5.894	<.001
Mother's education						1.403 (.383)	.109	3.662	<.001
Constant						−3.057 (1.123)		−2.721	.007
Adjusted R^2^									.232
N									911

^1^ Controlled for mother's education, breastfeeding, mother's age at delivery, infant's gender, and older child/children in the family.

^2^ Using backward stepwise selection method.

Next, we studied the factors related to sleep onset latency (Table 4). Based on the preliminary regression analysis, ten statistically significant predictors, alongside with control variables, were carried forward into multivariable analysis. The backward stepwise method resulted in a model that retained seven variables, of which only parenting stress (*β* = .072, *p* = .015), active (*β* = .291, *p* < .001) and passive (*β* = .120, *p* < .001) physical soothing styles, and active recreationally oriented family environment (*β* = ‐.070, *p* = .015) were statistically significant. The first three were connected to longer and the fourth to shorter sleep onset latency. Three of the predictors did not reach significance but remained in the final model based on model selection criteria. For this model (F(7, 1084) = 25.980, *p* < .001), the coefficient of determination was 13.8%.

**TABLE 4:** Results from pre‐screening regression analyses (one main predictor with covariates) and final multivariable regression model for sleep onset latency (BISQ).

	Regression with one main predictor^1^					Multivariable regression^2^			
Predictor	B (SE)	*β*	*t*	*P*	*R* ^2^	B (SE)	*β*	*t*	*p*
Parenting stress	.015 (.003)	.163	5.429	**<.001**	.033	.006 (.003)	.072	2.442	.015
Active−physical soothing style	.052 (.005)	.325	11.354	**<.001**	.108	.046 (0.005)	.291	10.108	<.001
Passive−physical soothing style	.050 (.010)	.152	5.182	**<.001**	.029	.039 (.009)	.120	4.217	<.001
Authoritative parenting style	−.131 (.053)	−.073	−2.457	**.014**	.012				
Authoritarian parenting style	−.086 (.065)	−.043	−1.314	.189	.008				
Permissive parenting style	.255 (.071)	.106	3.589	**.001**	.018				
Cohesion (FES)	−.066 (.022)	−.088	−2.944	**.003**	.014				
Expressiveness (FES)	−.082 (.022)	−.112	−3.781	**<.001**	.019	−.037 (.021)	−.050	−1.708	.089
Conflict (FES)	.032 (.017)	.061	1.930	.054	.010				
Independence (FES)	−.011 (.020)	−.016	−.525	.600	.007				
Achievement (FES)	.039 (.019)	.061	2.060	**.040**	.010				
Intellectual (FES)	−.014 (.015)	−.030	−.965	.335	.007				
Active−Recreational (FES)	−.045 (.014)	−.097	−3.255	**.001**	.016	−.032 (.013)	−.070	−2.439	.015
Moral (FES)	.005 (.014)	.011	.372	.710	.007				
Organization (FES)	−.056 (.015)	−.109	−3.682	**<.001**	.018	−.025 (.015)	−.050	−1.720	.086
Control (FES)	−.022 (.019)	−.041	−1.160	.246	.008				
Older child/children						−.075 (.040)	−.053	−1.860	.063
Constant						2.172 (.203)		10.708	<.001
Adjusted R^2^									.138
N									1092

^1^ Controlled for mother's education, breastfeeding, mother's age at delivery, infant's gender, and older child/children in the family.

^2^ Using backward stepwise selection method.

Third, we studied the factors related to the number of nighttime awakenings (Table 5). Four statistically significant factors in the preliminary regression analysis, alongside with the control variables, were used in the multivariable regression analysis. Backward elimination regression was conducted to identify the most significant predictors of the number of nighttime awakenings. The final model included parenting stress (*β* = .161, *p* < .001), active (*β* = .160, *p* < .001) and passive (*β* = .082, *p* < .004) physical soothing styles, breastfeeding (*β* = .157, *p* < .001), an older child or children (*β* = 0,067, *p* = 0,020); and the mother's education (*β* = 0,053, *p* = 0,071). The latter two were not statistically significant predictors for the number of nighttime awakenings but remained in the final model based on model optimization criteria. The final model was statistically significant (F(6, 1085) = 24.926, *p* < .001), explaining 11.6% of the total variation in nighttime awakenings

**TABLE 5:** Results from pre‐screening regression analyses (one main predictor with covariates) and final multivariable regression model for number of awakenings at night (BISQ).

	Regression with one main predictor^1^					Multivariable regression^2^			
Predictor	B (SE)	*β*	*t*	*P*	*R* ^2^	B (SE)	*β*	*t*	*p*
Parenting stress	.012 (.002)	.196	6.743	**<.001**	.089	.010 (.002)	.161	5.556	<.001
Active−physical soothing style	.021 (.003)	.1998	6.773	**<.001**	.088	.017 (.003)	.160	5.402	<.001
Passive−physical soothing style	.025 (.006)	.112	3.864	**<.001**	.063	.018 (.006)	.082	2.868	.004
Authoritative parenting style	−.023 (.036)	−.019	−.641	.522	.051				
Authoritarian parenting style	.004 (.044)	.003	.102	.919	.051				
Permissive parenting style	.139 (.048)	.085	2.911	**.004**	.058				
Cohesion (FES)	−.015 (.015)	−.029	−.991	.322	.052				
Expressiveness (FES)	−.021 (.015)	−.042	−1.449	.148	.052				
Conflict (FES)	.021 (.011)	.057	1.830	.068	.054				
Independence (FES)	−.020 (.014)	−.044	−1.449	.148	.052				
Achievement (FES)	−.010 (.013)	−.022	−.757	.449	.051				
Intellectual (FES)	.011 (.010)	.032	1.077	.282	.052				
Active−Recreational (FES)	.008 (.009)	.026	.889	.374	.051				
Moral (FES)	.011 (.009)	.034	1.145	.252	.052				
Organization (FES)	−.009 (.010)	−.027	−.910	.363	.051				
Control (FES)	.004 (.013)	.012	.33	.739	.051				
Older child/children						.065 (.028)	.067	2.338	.020
Breastfeeding						.159 (.030)	.157	5.235	<.001
Mother's education						.056 (.031)	.053	1.806	.071
Constant						.173 (.087)		1.997	.046
Adjusted R^2^									.116
N									1092

^1^ Controlled for mother's education, breastfeeding, mother's age at delivery, infant's gender, and older child/children in the family.

^2^ Using backward stepwise selection method.

Finally, we studied which factors explained the time that the infant spent awake at night (Table 6) using the same procedure as previously. The final model included parenting stress (*β* = .178, *p* < .001), an active physical soothing style (*β* = .085, *p* = .006), the mother's age (*β* = .080, *p* < .010), and breastfeeding (*β* = .097, *p* = .001). All of these predicted a longer time the infant spent awake at night. The passive physical soothing style and older child/children in the family were included in the final model but did not show statistical significance. The coefficient of determination for the final model was 6.7%, and was statistically significant (F(6, 1057) = 13.732, *p* < .001).

**TABLE 6:** Results from pre‐screening regression analyses (one main predictor with covariates) and final multivariable regression model for awake at night (BISQ).

	Regression with one main predictor^1^					Multivariable regression^2^			
Predictor	B (SE)	*β*	*t*	*p*	*R* ^2^	B (SE)	*β*	*t*	*p*
Parenting stress	.030 (.005)	.199	6.659	**<.001**	.062	.027 (.005)	.178	5.885	<.001
Active−physical soothing style	.033 (.008)	.124	4.041	**<.001**	.038	.023 (.008)	.085	2.775	.006
Passive−physical soothing style	.046 (.017)	.083	2.737	**.006**	.030	.032 (.016)	.057	1.920	.055
Authoritative parenting style	−.010 (.093)	−.003	−.107	.914	.023				
Authoritarian parenting style	−.197 (.113)	−.059	−1.742	.082	.026				
Permissive parenting style	.366 (.123)	.090	2.969	**.003**	.031				
Cohesion (FES)	−.058 (.039)	−.047	−1.511	.131	.025				
Expressiveness (FES)	−.092 (.038)	−.075	−2.450	**.014**	.028				
Conflict (FES)	.029 (.029)	.032	.980	.327	.024				
Independence (FES)	−.032 (.035)	−.029	−.906	.365	.023				
Achievement (FES)	.030 (.033)	.028	.929	.353	.024				
Intellectual (FES)	−.028 (.026)	−.034	−1.094	.274	.024				
Active−Recreational (FES)	−.021 (.024)	−.026	−.861	.389	.023				
Moral (FES)	−.016 (.024)	−.021	−.682	.495	.023				
Organization (FES)	−.031 (.026)	−.036	−1.186	.236	.024				
Control (FES)	−.010 (.033)	−.011	−.311	.756	.023				
Mother's age						.021 (.008)	.080	2.569	.010
Older child/children						−.132 (.074)	−.055	−1.782	.075
Breastfeeding						.244 (.077)	.097	3.191	.001
Constant						.367 (.321)		1.142	.254
Adjusted R^2^									.067
N									1064

^1^ Controlled for mother's education, breastfeeding, mother's age at delivery, infant's gender, and older child/children in the family.

^2^ Using backward stepwise selection method.

## 4 DISCUSSION

In this study, we explored which parenting factors increase the risk of infant sleeping problems. We examined the connection between infants’ problematic sleep and family environmental factors experienced mainly by mothers. Problematic sleep was measured using the ISQ severity score along with single aspects describing the quality of sleep: sleep onset latency, number of awakenings, and time spent awake at night. Parenting styles, family climate, parenting stress, and preferred soothing methods were included as predictors in the statistical models representing the family environment. The results indicated several associations between infant sleep and environmental factors. Most importantly, infant sleep problems were related to an active physical soothing style and parenting stress, while many of the studied family environmental factors did not show statistically significant connections with infant sleep—parenting styles and most of the family climate dimensions, except for the active recreational and control orientation.

The results indicated a noticeable link between elevated parenting stress and infant sleep problems in all four models, in line with previous research (Tyler et al., 2019; Meltzer & Mindell, 2007; Doo & Wing, 2006; Hodge et al., 2013; Zengin Akkus & Bahtiyar‐Saygan, 2022). Although parenting stress acted as a predictor in the statistical models, the cross‐sectional design did not allow for causal interpretations of the findings. It is possible that the direction of the relationship is opposite to what our models suggest: Infants’ sleeping problems cause fatigue and stress to parents. It is obvious that infant sleeping problems are stressful for parents. Children's sleep problems can cause disrupted sleep for mothers, which can increase maternal stress (Meltzer & Mindell, 2007). Parenting stress may also be a result of mothers feeling responsible for their children's sleeping problems (Zengin Akkus & Bahtiyar‐Saygan, 2022).

Another possible interpretation of this result is that stressed mothers perceive their infants’ sleep as more problematic and report more awakenings at night than really occur. Sinai & Tikotzky (2012) found that parents who reported higher levels of parenting stress also described their infants’ sleep as more problematic, even though there was no relationship between infants’ sleep patterns and parenting stress, as assessed from sleep diaries. Similar findings were reported by Tikotzky et al. (2021), wherein maternal emotional distress (including parenting stress symptoms) correlated with maternal reports of problematic infant sleep but not with actigraphy measures. Based on their findings, Tikotzky et al. (2021) recommended that clinical interventions for emotionally distressed mothers and their infants with sleeping problems should prioritize maternal sleep‐related cognitions and interpretations rather than attempting to directly alter infant sleep patterns.

Nevertheless, the bidirectional nature of the connection between stress and sleep (Sadeh et al., 2010) must be considered equally. There are suggestions that maternal stress can transfer to the infant (Waters et al., 2014). Parenting stress can also influence children's sense of security via parent–child interactions, and stress or insecurity may be reflected in sleep problems (Thome & Skuladottir, 2005). Also, the connection between parenting stress and increased use of bedtime interactions has been reported (Millikovsky‐Ayalon et al., 2015). Maternal stress correlated with both active and passive physical soothing styles in our study. As the findings between stress and sleeping problems allow for various interpretations, they underscore the necessity for further empirical investigation.

In addition, we found that soothing methods were linked to infant sleep. As expected, the more the parents used active physical comforting, the more problematic infant sleep was (i.e., the higher the infant's sleep problem severity score, the longer it took the infant to fall asleep; the higher the number of night awakenings, the longer the infant was awake at night). Active physical comforting was connected to problematic sleep slightly more than maternal stress in some of the models. The relationship between passive physical comforting and problematic sleep was also clear, but was not as strong as active physical comforting. These results regarding active physical soothing methods fall well in line with previous studies’ findings (Morrell & Cortina‐Borja, 2002; Sadeh et al., 2009, 2010; Morrell & Steele, 2003; Adams et al., 2022). Too active physical involvement at bedtime probably hinders the development of self‐soothing skills (Zreik et al., 2021; Adams et al., 2022). Adams et al.’s (2022) study supports this assumption: infants whose parents used low‐ rather than high‐stimulus soothing strategies were more able to fall back to sleep on their own without parental involvement. They observed infant sleep using actigraphy, which strengthened the results, although the sample size was small. However, Quante et al. (2022) reported that bottle‐feeding at bedtime was associated with more continuous sleep, and exclusive breastfeeding was positively linked to better sleep quantity and quality. Feeding is considered an active physical soothing method in our study, and breastfeeding is associated with more sleeping problems (Sadeh et al., 2010).

However, the transactional model of sleep and parenting and bidirectional relationships (Sadeh et al., 2010) must also be considered here. It is not entirely certain that soothing methods have direct effects on infant sleeping problems. Parents can adjust their methods based on how their infants react to them (El‐Sheikh & Kelly, 2017). Infant temperament or sleep patterns may determine what kind of soothing method is required, and infants with difficult sleep patterns need more involvement from their parents (Morales‐Munoz et al., 2020; Sadeh et al., 2010; Zreik et al., 2021). This reverse direction of effects, from infant sleep to parental involvement, was examined by Matzliach et al. (2025), yet the findings provided only limited support, suggesting that infant sleep problems at 4 months predicted, surprisingly, decreased parental bedtime involvement over the following months (Matzliach et al., 2025). The relationship between soothing styles and sleep problems is more likely influenced by prevailing habits and norms and is culturally specific (Zreik et al., 2021). Active physical comforting may not be associated with sleep problems in cultures where cosleeping is the norm. However, bedsharing and cosleeping have been found to be associated with shorter sleep duration and more frequent night awakenings in infants (Hysing et al., 2014).

The findings suggest that certain dimensions of the family climate may play a minor role in shaping infant sleep. The family climate was measured at the 3‐month time point, and sleep at the 8‐month time point. However, since family climate is considered a relatively stable trait, we do not consider the difference in timing between the FES and the sleep measures as problematic. Sleep was significantly associated with control and active recreational dimensions in the family, even though the relationship was very modest. Our results did not support our hypothesis and were inconsistent with previous research, as we found no statistically significant association between conflict and sleep. The control is a system maintenance dimension in the FES, reflects internal family functioning (Moos & Moos, 2009), and represents the extent to which a family has established rules and procedures. Higher levels of control were associated with higher sleep problem severity scores. Control measured with FES has been linked to both positive and negative outcomes, particularly in adolescence. These mixed results are understandable, because in general the impact of control is not straightforward and depends on the emotional climate, especially the warmth of the family environment (Doo and& Steinberg, 1993). The influence of control also emerges as a central feature in parenting styles: authoritative parenting combines high warmth with high control, authoritarian parenting shows high control while warmth is minimized, and permissive parenting offers warmth without consistent control (Darling & Steinberg, 1993). In our study, the authoritarian parenting style correlated positively and the authoritarian correlated negatively with the control dimension. The permissive parenting style had no statistically significant correlation with the control dimension.

Although studies utilizing the FES scale to explore the link between control‐oriented family climate and children's sleep are lacking, there is some evidence to suggest that excessive control as a parenting practice, measured by instruments other than FES, is associated with poorer sleep outcomes in children (Philips et al., 2014). This association may be explained by several factors. For example, parents of control‐orientated families might be more stressed, which reflects their interaction with their child. Control and parental stress showed a weak correlation in our study. Another possible reason is that high parental control may impair sleep if bedtime practices are not adapted to the child's developmental needs.

Active recreational orientation is the FES's personal growth dimension (Moos & Moos, 2009). According to Moos & Moos (2009), personal growth dimensions mostly reflect the connection between the family and the larger social context, referring to how much the family participates in social and recreational activities. There was a connection between more active, recreationally oriented families, and shorter sleep onset latency. An active recreational orientation has been linked to positive developmental outcomes in older children and adolescents, for example, to the mental well‐being of adolescent girls (Maheshwari et al., 2020). To our knowledge, previous studies have not reported connections between active recreationally oriented families and infant sleep. In active, recreationally oriented families, free time is emphasized, and they are socially active (Moos & Moos, 2009) and tend to engage in frequent joint leisure activities. Family leisure involvement has been associated with greater satisfaction with family life (Zabriskie & McCormick, 2003) and lower stress (Bedini et al., 2018). It can be assumed that this has favorable effects on the safety of the developmental environment, parent–child interaction, the child's emotional well‐being, and through these, on the child's sleep. Active daytime routines may also support the development of healthy sleep‐wake rhythms and help infants fall asleep more easily.

Further research is needed, for example, to investigate the mediating role of parenting stress between infant sleep and an active recreational orientation. Despite the statistical significance of control and active recreational orientations, their beta values were very small, suggesting that these predictors have limited explanatory power. It is important to note that statistical significance does not necessarily imply practical relevance, especially in large samples in which even trivial associations may yield low p‐values.

Contrary to our expectations, of the three parenting styles, none had a connection to infant sleep quality based on the multivariable regression analyses. The finding that permissive parenting was not associated with child sleep contradicts previous research. However, previous findings on the association between permissive parenting and child sleep problems have primarily focused on slightly older children. The finding that the authoritarian parenting style was not associated with sleeping problems aligns with Tyler et al.’s (2019) findings. Usually, the authoritarian parenting style is associated with negative child development outcomes, especially among older children (Baumrind et al., 2010). It is, however, understandable that the authoritarian parenting style did not predict children's sleep problems in this age group (< 1 years). Authoritarian parents tend to be stricter and set rules and boundaries for their children (Tyler et al., 2019; Baumrind et al., 2010). These may include establishing strict bedtime routines and not responding as easily to infant waking and crying (Tyler et al., 2019; Rodriguez et al., 2025). However, the authoritarian parenting style was also not found to positively influence children's sleep, in line with Tyler et al.’s (2019) findings. The authoritative parenting style has been associated with numerous positive child outcomes (Baumrind, 1966). It would therefore be reasonable to expect a similar association with children's sleep as well (Tyler et al., 2019). Given the children's early developmental stage, parenting style may influence interaction more noticeably at a later age. Baumrind's classification of parenting styles has been criticized, for example, for its centration in Euro‐American frames. Also, this classification may not fully capture the characteristics of Finnish families. For example, authoritarian parenting is less prevalent in Finland today than in the past (Kivijärvi et al., 2009).

## 5 LIMITATIONS

The results of this study must be evaluated through the following limitations. The first concerns the measurement of parenting styles. Most of the parents in this study were clearly authoritative, while a highly authoritarian parenting style was rare. This point should be considered when assessing the findings’ generalizability. Second, the PSDQ questionnaire included items concerning the parenting of older children. These items were left out of the study questionnaires to avoid participant confusion. However, the Cronbach's alphas of the PSDQ subscales constructed by the remaining items showed adequate reliability. We emphasize that it was highly important to include a measure of parenting style in this study because there is a lack of research on the role of parenting styles, especially that measured using the PSDQ, on infant sleep.

Third, when interpreting our results, we must pay attention to low coefficients of determination. Especially in the model concerning time spent awake at night, family environmental factors explained only 6.7% of the total variation in infant sleep. However, the factors were related to infant sleep at a statistically significant level, according to F‐tests (*p* < .001 for all models), suggesting that they have predictive power. Low beta coefficients should also be taken into account, as they indicated that the included predictors may have only modest effects on the outcome.

Fourth, although the assessment of infant sleep based on subjective reports is evidently the most used method among studies, it has some deficiencies. It has been reported that parents tend to underestimate the frequency of night awakenings and other sleep parameters (McNamara et al., 2003; Tikotzky & Sadeh, 2001; Vaughn et al., 2011; Scher, 2001). However, according to McNamara et al. (2003), the overall pattern of night awakenings over time is not distorted by maternal reports. Nevertheless, more objective measurements of sleep, such as actigraphy, could have produced additional information (Bordeleau et al., 2012). Moreover, not only sleep measures but all other variables relied on subjective reporting, which may increase the risk of shared method variance. For example, external observations of parenting and family factors could provide more precise information about these connections.

Fifth, the ISQ was originally developed to assess the sleep of 12–18‐month‐old children, but the children in our sample were 8 months old. However, the items were simple, and their face validity was high, even among infants younger than 1 year old. The sixth limitation of our study was the cross‐sectional nature of the data analysis. Causal interpretations of the connections between family environment and infant sleep cannot be made.

Finally, due to the low reliability estimates of the PIBBS, two out of five composite variables representing bedtime strategies had to be excluded from the regression analysis. The reliability estimates of the remaining two—active and passive physical soothing styles—were relatively low. However, some methodological literature suggests that an alpha value of .50 may still be acceptable in certain contexts, particularly when dealing with short scales or exploratory research. However, it should be acknowledged that the relatively low internal consistency of the scales may have implications for the interpretation of the results.

### 5.1 Further research

Based on our findings, there is a need for further studies. In our study, the mothers were the main informants of the family environment. Studying fathers’ perspectives would add value to the body of research. Future research would also benefit from objective data collection, such as video recordings, direct observation, sleep diaries, and actigraph registration. From further longitudinal studies, it would be interesting to see how these factors are linked with sleep development, how they are necessary to determine if family environmental factors predict sleeping problems in small children, and to consider the bidirectional effects of these factors and infant sleep. Moreover, further research is required to specifically examine the direct and indirect connections of parenting style and family climate with infant sleep. Although parenting style was not found to have a direct effect on infant sleep, it is nevertheless important to examine whether parenting style mediates the relationship between parenting stress and infant sleep, and it may also be relevant for children's sleep quality beyond infancy. It is also important to investigate what explains the relationship between a control‐oriented family climate and infant sleeping problems. Is this because such a family climate is somewhat stressful for infants, which manifests as sleeping problems? How significant is the influence of infant temperament?

## 6 CONCLUSION

In conclusion, this study not only provided results that support previous findings about the relationship between family environment and children's sleep but also offered some new information. Based on our results, maternal stress, physical soothing styles, and a control‐oriented family climate are linked to a worse quality of infant sleep, while an active‐recreational family climate dimension is connected to a better quality of infant sleep. The question of whether parenting factors might help prevent infant sleeping problems is important for parents and clinicians. Although further research is needed to answer this question, our findings make a worthwhile contribution to knowledge about the links between family environment and children's sleep, and these results should be taken into consideration when planning support for families who seek help with infant sleeping problems.

## CONFLICT OF INTEREST STATEMENT

The authors declare that this research has no commercial or financial relationship that could be construed as a potential conflict of interest.

## Data Availability

The data that support the findings of this study are available on request from the corresponding author. The data are not publicly available due to privacy or ethical restrictions.
